# Differential regulation of aggressive features in melanoma cells by members of the miR-17-92 complex

**DOI:** 10.1098/rsob.140030

**Published:** 2014-06-11

**Authors:** Eyal Greenberg, Steven Hajdu, Yael Nemlich, Ronit Cohen, Orit Itzhaki, Jasmine Jacob-Hirsch, Michal J. Besser, Jacob Schachter, Gal Markel

**Affiliations:** 1 Sheba Medical Center, Ella Institute of Melanoma, Ramat Gan, Israel; 2 Clinical Microbiology and Immunology, Sackler School of Medicine, Tel Aviv University, Tel Aviv, Israel; 3 Cancer Research Center, Ramat Gan, Israel; 4 Talpiot Medical Leadership Program, Sheba Medical Center, Ramat Gan, Israel

**Keywords:** miR-17, miR-20a, melanoma, miR-17-92 cluster, proliferation, differential regulation

## Abstract

The various roles of microRNAs (miRNAs) in controlling the phenotype of cancer cells are the focus of contemporary research efforts. We have recently shown that miR-17 directly targets the ADAR1 gene and thereby enhances melanoma cell aggressiveness. miR-17 and miR-20a belong to the miR-17/92 complex, and their mature forms are identical except for two non-seed nucleotides. Nevertheless, here we show that these two miRNAs carry markedly different effects on melanoma cells. A strong positive correlation was observed between the expression of miR-17 and miR-20a among various melanoma cultures. Luciferase assays showed that miR-17 but not miR-20a directly targets the 3’ untranslated region of the ADAR1 gene. Ectopic expression of these miRNAs in melanoma cells differentially alters the expression of five exemplar TargetScan-predicted target genes: ADAR1, ITGB8, TGFBR2, MMP2 and VEGF-A. Whole-genome expression microarrays confirm a markedly differential effect on the transcriptome. Functionally, over-expression of miR-20a but not of miR-17 in melanoma cells inhibits net proliferation *in vitro*. The differential functional effect was observed following ectopic expression of the mature miRNA or of the pre-miRNA sequences. This suggests that the two non-seed nucleotides dictate target sequence recognition and overall functional relevance. These miRNAs are clearly not redundant in melanoma cell biology.

## 2. Introduction

Melanoma is a cancer that develops from melanocytes located predominantly in the skin, but also found in the eyes, ears, gastrointestinal tract, and oral and genital mucous membranes [1]. While melanoma accounts for nearly 4% of all skin cancers, it causes 75% of skin cancer-related deaths worldwide and is considered to be the most common fatal malignancy of young adults [2]. Although new lines of targeted therapy [3–7] and immunotherapy [8–11] were introduced lately, durable responses are not common as it is hard to target the elusive metastatic phenotype.

Adenosine deaminase acting on RNA 1 (ADAR1) enzyme mediates adenosine to inosine (A-to-I) RNA editing at the post-transcriptional level. It was previously reported that many solid tumours exhibit lower A-to-I RNA editing [12], but the mechanistic and functional significance have remained obscure. Recently, we reported that ADAR1 is frequently downregulated during the metastatic transition by using progression tissue microarrays. We found that ADAR1 regulates the malignant phenotype of melanoma cells by controlling the biogenesis pathway of microRNAs (miRNAs) in an RNA-editing independent manner, and that metastatic melanoma cells silence ADAR1 by over-expressing miR-17 and miR-432, which both directly target the ADAR1 transcript [13].

MiRNAs are non-coding small sequences of RNA molecules of 19–22 nucleotides that function as specific post-transcriptional regulators of gene expression [14]. Once processed from their distinctive hairpin transcripts and loaded into the Argonaute protein of the silencing complex, the miRNA pair with the 3’ untranslated region (3'UTR) of the target mRNA to direct post-transcriptional repression in multiple pathways. Perfect pairing between a miRNA and its target site induces endonucleolytic cleavage by Argonaute, leading to rapid degradation of the mRNA [15,16]. Partial pairing of the miRNA complex to target 3′UTR sites can result in de-adenylation of the mRNA [17]. The miR-induced silencing complex (miRISC) can also induce translational repression by blocking its initiation [18,19], by promoting ribosome drop-off [20] or by stimulating proteolysis of the nascent peptide [21]. miRNAs have also been shown to upregulate target expression under certain conditions through a mechanism that involves Argonaute and fragile X mental retardation protein 1 (FMR1). Imperfect pairing of the 5′ end of the miRNA to a target is sometimes compensated for by extensive 3′ end interactions, as are evident in the lethal 7 (let-7) miRNA target site in the abnormal cell lineage 41 (lin-41) 3′UTR in *C. elegans* [22].

MiRNAs have been found to be involved in early development, cell differentiation, cell cycle, apoptosis, angiogenesis and tumour progression [23]. Various regulatory roles for miRNAs have been directly implicated in cancer development, progression and metastasis *in vitro*, *in vivo* and in patients [14,24]. Suppressive roles for miRNAs were first described for miR-15 and miR-16, which are preferentially deleted and downregulated in B-cell chronic lymphocytic leukaemia [25,26]. Many additional suppressive miRNAs, which are encoded in cancer-associated chromosomal fragile sites, have been described since then [27,28]. Conversely, miRNAs have also been identified as potential oncogenes. A well-studied, potentially oncogenic cluster of miRNAs is the polycistron miR-17/92, which contains seven miRNAs and is frequently over-expressed in various tumours [23,29]. Over-expression of miRNAs derived from this cluster facilitates cancer, and promotes migration and invasion in several malignancies [23,29]. It should be noted that in some cases, a lineage-specific gene expression profile might dictate differential regulatory roles for a specific miRNA across different cell types. Thus, the definition of miRNA as a tumour suppressor or an oncogene should be made in the context of specific cell types, as evident in the cases of miR-31 and miR-20a [30–42].

Here we show that miR-17 and miR-20a, both members of the miR-17/92 cluster with identical seed sequences, exhibit differential molecular and phenotypic effects, as well as predicted affected biological pathways, in melanoma cell lines. We show that distinct nucleotides placed outside the seed region, and found both in the pre and the mature miRNA, account for this phenomenon. These findings imply an additional level of regulation by miRNAs.

## 3. Results

### 3.1. Differential effect of miR-17-5p and miR-20a on 3′UTR of ADAR1

**Figure 1.**
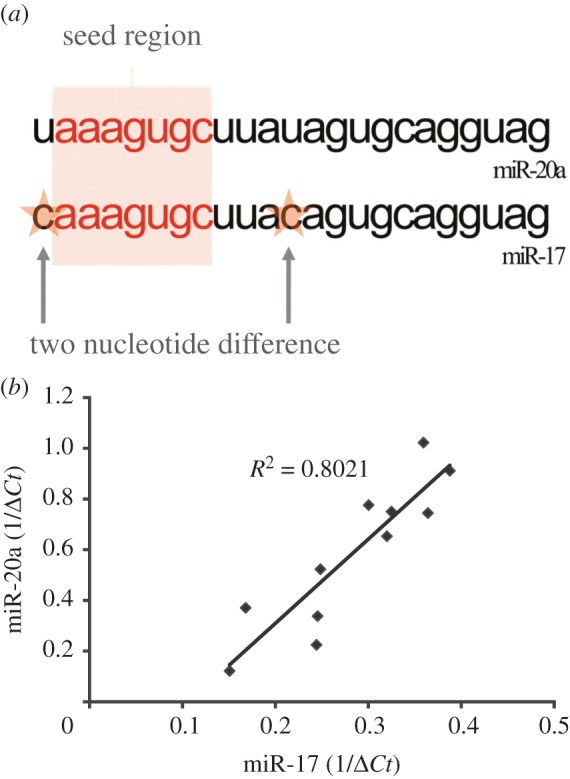
hsa-miR-17 versus hsa-miR-20a. (*a*) Same cluster mature miR-17 and miR-20a nucleotide composition. (*b*) miR-17 versus miR-20a expression in 11 melanoma cell cultures.

Previous studies with isogenic melanoma cells lines suggest over-expression of miR-17-5p in aggressive melanoma, which enhances proliferation of melanoma cells in controlled *in vitro* experiments [43]. This effect could be at least partially explained by direct targeting of ADAR1 expression [13]. miR-20a is another member of the miR-17/92 cluster. The mature form of miR-20a is identical to miR-17-5p, except for two nucleotides outside the seed region (figure 1*a*). Accordingly, bioinformatics predicts for both miRNAs the same target genes, since they share the same seed region (electronic supplementary material, table S1). In agreement with their genomic location within the miR-17/92 polycistron, the expression of both miRNAs exhibits a strong correlation in 11 melanoma cultures tested (figure 1*b*; *R*^2^ = 0.8021).

**Figure 2.**
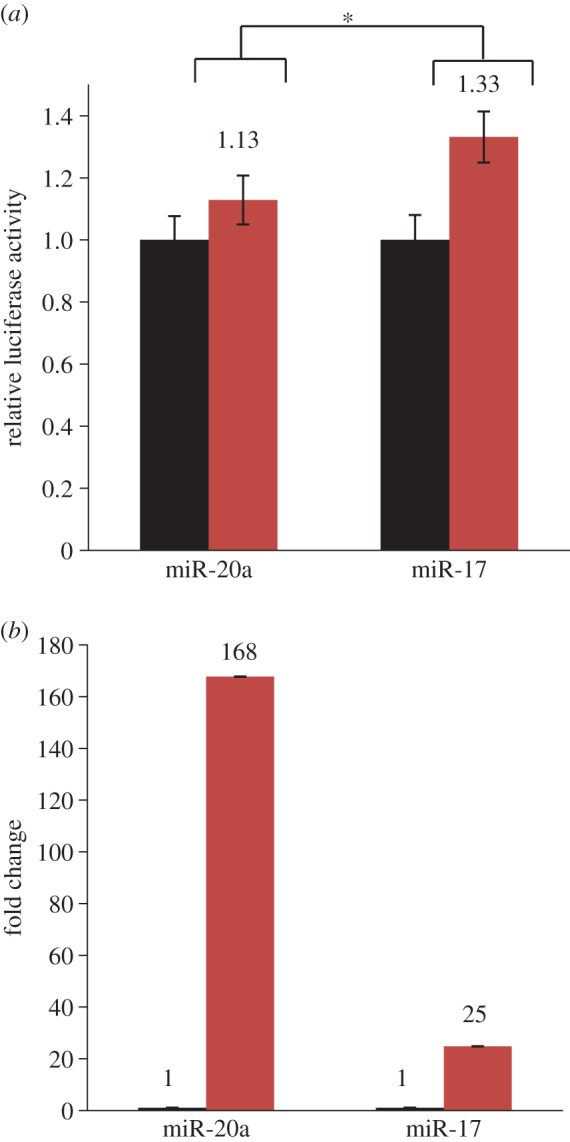
miR-17 versus miR-20a differential effect on ADAR 3′UTR. (*a*) Dual luciferase assay conducted on ADAR 3*′*UTR as representative target gene. The assay included forced expression of miR-17 and miR-20a in HEK 293t cell line. The miRNAs transfected cells were separately co-transfected with the dual luciferase psicheck2 vector included the mutated 3*′*UTR (red bars) of interest compared with the wild-type 3*′*UTR (black bars). The upstream Renilla luciferase activity was measured and normalized to the firefly constitutive luciferase activity. Asterisk denotes *p*-value of <0.05. (*b*) Verification of miR-17-5p and miR-20a over-expression in HEK 293t transfectants, as compared with scrambled-transfected cells. The *y*-axis denotes fold change above scrambled-transfected cells (black bars, control; red bars, over-expression).

In order to study the potential redundancy of miRNAs within this cluster, repression of ADAR1 expression in the dual luciferase experimental system was tested with miR-20a and miR-17-5p. HEK-293T cells were co-transfected with mature miRNA or scrambled sequence, together with dual luciferase psicheck2 vector containing the wild-type 3'UTR of ADAR1, or one carrying abolishing mutations in the miR-17-5p binding site. In line with our previous results [13], miR-17-5p directly targets the 3'UTR of ADAR1 (figure 2*a*). Surprisingly however, miR-20a displayed a significantly weaker effect on ADAR1 expression (figure 2*a*), even though it was over-expressed by more than sixfold stronger than miR-17-5p (figure 2*b*). This result provides the first clue that there might be no redundancy among miR-17-5p and miR-20a.

### 3.2. Differential effect of miR-17-5p and miR-20a on gene expression

**Figure 3.**
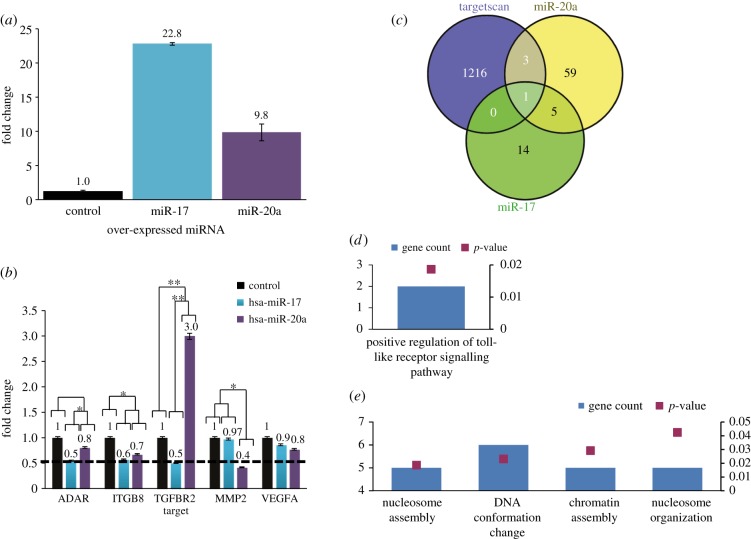
Differential effect on TargetScan-predicted targets of miR-17 versus miR-20a transfected melanoma cell line. (*a*) Verification of miR-17-5p and miR-20a over-expression in PAG transfectants, as compared with mock-transduced cells. The *y*-axis denotes fold change above mock-transduced cells. (*b*) Real-time PCR performed on miR-17 and miR-20a TargetScan-predicted target genes. The *y*-axis denotes fold change above mock-transduced cells. Results are of three experimental replicates. Statistical analysis was performed with ANOVA. Asterisks (*) and (**) denote *p*-values of <0.05 and 0.01, respectively. (*c*) Venn diagram of TargetScan's miR-17/20a predicted targets with microarrays gene expression profile of miR-20a- and miR-17-transduced PAG cells. (*d*) GO: biological process informatic analysis derived from miR-17 downregulated genes. (*e*) GO: biological process informatic analysis derived from miR-20a downregulated genes.

The differential effect of miR-17-5p and miR-20a on gene expression in melanoma cells was further studied by focusing on four TargetScan-predicted target genes: ITGB8, TGFR2, MMP2 and VEGF-A. These genes are known to play significant roles in cancer progression and metastasis [44–53]. ADAR1 served as a positive control. C81–61 melanoma cells (poorly aggressive cells, PAG) were transfected with miR-17-5p, miR-20a or empty vector and RNA was collected 3 days post-transduction. Expression of the transduced miRNAs was verified (figure 3*a*). As expected from the luciferase experiments (figure 2*a*), ADAR1 expression was downregulated by miR-17-5p but not by miR-20a (figure 3*b*). Remarkably, two additional transcripts (TGFBR2 and MMP2) exhibited marked differential expression between the miR-17-5p- and miR-20a-transduced cells (figure 3*b*). TGFR2 was downregulated by miR-17-5p and upregulated by miR-20a, while MMP2 was downregulated only by miR-20a (figure 3*b*). ITGB8 exhibited questionable regulation by both miRNAs, while VEGF-A was entirely unaltered by both. While the stronger expression of miR-17 compared with miR-20a (figure 3*a*) must be taken into consideration when interpreting downstream events, it fails to explain the differential regulation of TGFBR2 and MMP2 (figure 3*b*). Next, the effect of each miRNA on the transcriptome was tested by whole-genome oligonucleotide microarrays. A value of 1.5-fold change was predetermined as cut-off for altered expression. A total of 20 and 68 genes were significantly downregulated following over-expression of miR-17-5p or miR-20a, respectively. Strikingly, only one gene was in common when overlapped with TargetScan prediction (figure 3*c*; electronic supplementary material, table S2). Moreover, bioinformatics analysis of the downregulated genes revealed that each miRNA affects different biological functions (figure 3*d,e*). These combined results imply that miR-17-5p and miR-20a are not redundant and are expected to exert different functional effects.

### 3.3. miR-17-5p and miR-20a exert opposite effects on proliferation

**Figure 4.**
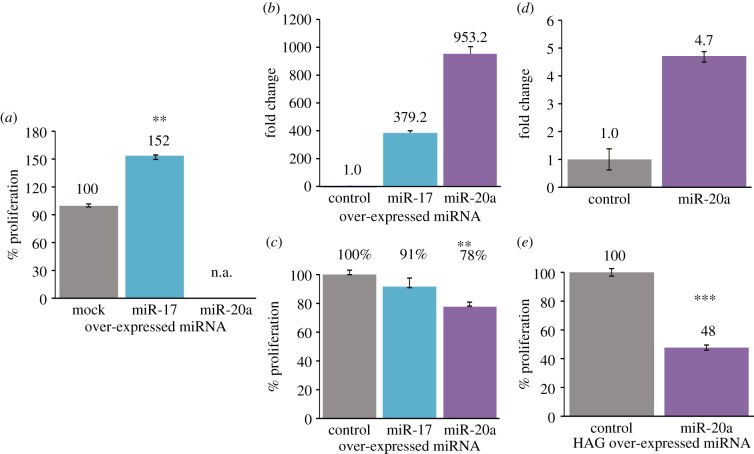
miR-20a effect on proliferation of melanoma cell line. (*a*) Net proliferation of the PAG-transduced cells was quantified with standardized XTT test. The number of cells was determined 48 h after seeding. The number of mock-transduced cells was determined as 100%. The figure shows a representative experiment out of three performed. Asterisk (**) denotes *p*-value of <0.001. (*b*) Verification of miR-17 and miR-20a over-expression in mimetic PAG-transfected cells, as compared with scrambled sequence transfected cells. The *y*-axis denotes fold change above mock-transduced cells. (*c*) Net proliferation of the miR-mimetic PAG-transfected cells was quantified with standardized XTT test. The number of cells was determined 48 h after seeding. The number of mock-transduced cells was determined as 100%. The figure shows a representative experiment out of three performed. Asterisk (**) denotes *p*-value of <0.01. (*d*) Verification of miR-20a over-expression in HAG-transduced cells, as compared with mock-transduced cells. The *y*-axis denotes fold change above mock-transduced cells. (*e*) Net proliferation of the HAG-transduced cells was quantified with standardized XTT test. The number of cells was determined 48 h after seeding. The number of mock-transduced cells was determined as 100%. The figure shows a representative experiment out of three performed. Asterisk (***) denotes *p*-value of <0.01.

PAG melanoma cells were stably transduced with miR-17-5p and miR-20a constructs, as well as with an empty vector as control. The over-expression of miR-17-5p and miR-20a was verified (figure 3*a*). miR-17-5p enhanced the proliferation ability of PAG melanoma cells (figure 4*a*). By contrast, the miR-20a-transduced cells did not survive more than one week post-transduction and could not be accurately tested for proliferation (figure 4*a*). This observation suggests that miR-20a might induce a strong suppressive effect on the melanoma cells, the opposite from miR-17-5p. In order to further explore the potential effect of miR-20a on proliferation, PAG cells were transiently transfected with the RNA sequences of the mature miRNAs or a control sequence (figure 4*b*). A different effect on proliferation was still evident in this experimental system as well. Transient transfection of mature miR-17-5p did not exert a significant effect on proliferation when compared with control sequence, whereas transfection of miR-20a exerted a significant suppressive effect on PAG cell proliferation (figure 4*c*). The suppressive effect of miR-20a was further tested in the highly aggressive (HAG) C8161 cells. In agreement with the results depicted above, stable transduction of the miR-20a constructs into HAG cells (figure 4*d*) substantially inhibited proliferation as compared with mock-transduction (figure 4*e*). Collectively, these experiments show that there is no functional redundancy in melanoma between miR-17-5p and miR-20a, as they exert opposite functional effects.

## 4. Discussion

To date, miRNAs are known to regulate 3'UTRs through imperfect pairing to adjacently transcribed mRNAs. A mandatory full match of the mRNA targeted sequence to the recognition site (e.g. seed region) found between nucleotides 2–8 in the 5’ of the mature miRNA will contribute to efficient mRNA degradation, translational inhibition or a combination of the two. Imperfect pairing of the 5′ end of the miRNA to a target is sometimes compensated by extensive 3′ end interactions (e.g. compensatory sites). Here, we addressed the potential role of miR-20a as a negative regulator of ADAR1.

We hypothesized that miR-20a might serve as a direct regulator of ADAR1 due to its seed region, which is identical to that of miR-17, an already proven direct regulator of ADAR1 [13]. Indeed, target-predicting algorithms do not differentiate between miR-17 and miR-20a. Surprisingly, miR-20a failed to experimentally target ADAR1 (figures 2*a* and 3*b*). Furthermore, it exhibited different regulation effects from miR-17 on three other potential target genes tested by qPCR (figure 3*b*). Remarkably, comparative whole-gene microarray experiments demonstrated only one gene transcript to be a predicted target and downregulated by both miRNAs (figure 3*c*; electronic supplementary material, table S2), and, accordingly, that different biological functions are affected (figure 3*d,e*). These results collectively indicate that these two miRNAs are not redundant, as they facilitate significantly different biological effects. To date, most of the publications regarding the miR-17/92 cluster suggest an oncogenic role for its members [23,29,41]. Interestingly, miR-20a, which is a member of this cluster, is reported in the literature as an onco-miRNA in several malignancies, including cervical cancer, osteosarcoma cells and ovarian cancer [39,40,42], whereas in malignancies such as breast cancer, hepatocellular carcinoma and squamous cell carcinoma it serves as a tumour suppressor [32,37,38]. This might be indicative for a dual role of miR-20a only but not miR-17, which depends on the gene expression profile. It should be emphasized that our results suggest that miR-17 and miR-20a affect differently the *same* melanoma cells. To the best of our knowledge, this is the first evidence for different effects of miRNAs of the same cluster within the same cancer cells.

Molecularly, the mature miR-17 and miR-20a sequences differ in only two nucleotides, which are located outside of the seed region (figure 1*a*), but their pre-miRNA precursors are entirely different. To discern the source for differential effects, we conducted a series of experiments using pre-miRNA and mature sequences of both miRNAs, in two melanoma cell lines. In all experimental set-ups tested, differential cell regulation was still observed (figure 4). The different effects exerted by mature miR-17 and mature miR-20a immediately point to a potential role in target recognition for the two extra non-seed nucleotides. Brennecke *et al.* [54] showed that extra non-seed nucleotides (e.g. nucleotide 1 in 7-mer versus 6-mer of a mature miRNAas well as 3'UTR compensatory sites) enhance the efficiency of a miRNA molecule to target its complementary mRNA sequence. These observations corroborate our results as the variations between miR-17 and miR-20a fall in nucleotides number 1 and 12 of the mature miRNA sequence [54]. Moreover, Brennecke *et al.* demonstrated that members of a given miRNA family, sharing the same seed, might differ in their sensitivity to mRNA target, using 3’ UTR reporters of the pro-apoptotic gene GRIM, an identified miRNA target [55]. This gene contained K boxes in its 3'UTR that is complementary to the 5’ ends of the miR-2, miR-6 and miR-11 miRNA family [55,56]. These miRNAs share residues 2–8 but differ considerably in their 3’ regions. The site in the 3’ UTR was predicted to form a 6-mer seed match with all three miRNAs, but only miR-2 showed the extensive 3’ complementarity that they predicted would be needed for a 3’ compensatory site with a 6-mer seed to function. Indeed, only miR-2 was able to regulate the 3’ UTR reporter, whereas miR-6 and miR-11 were non-functional [54]. The miR-17/92 cluster comprises miRNAs that can be grouped based on the similarity of their seed regions (nucleotides 2–8). Jiang *et al.* demonstrated that inside primary CD4^+^ T cells, and despite the very high degree of homology within the MIR-17 family, miR-20a was not capable of performing any of pro-Th1 functions of miR-17, and miR-18 clearly exerted an antagonistic effect. A similar distinction was also observed between miR-19a and miR-19b. As already mentioned, in the mature miRNAs, few nucleotides differ between miR-17 and miR-20a/miR-18a, and these differences reside outside the seed region. Jiang *et al.* suspected that these subtle differences are sufficient to result in a significant affinity difference between the miRNAs and their targets to produce the observed differential targeting, and clearly showed the same differential effect observed in our study of miR-17 versus miR-20a on TGFBR2 supported by a luciferase reporter assay in the NIH3T3 cell line [57]. Alternatively, because the pre-miRNA loops also differ between miR-17 and miR-20a/miR-18a, Jiang *et al.* hypothesized it was possible that the targeting preference between them was caused partially by differences in their pre-miRNA loops. The basis for this hypothesis was a previous report that loop sequence of the pre-miRNA may participate in the process of target recognition, as distinct mRNA-targeting activities of miR-181a and miR-181c were largely determined by their divergent pre-miRNA loop sequences, but not by the one-nucleotide alteration in the mature miRNAs [58]. Regardless, their results did argue that the sequences outside the seed region could also be an indispensible component of the miR-targeting machinery [57].

In conclusion, despite the fact that miR-17 and miR-20a arise from the same cluster and have identical seed regions, they confer differential functional effects and target different genes in the same melanoma cells. The fact that miR-17-5p is commonly described as an onco-miRNA, while miR-20a was demonstrated in some cases as an onco-miRNA and in others as a suppressive-miRNA, corroborates our hypothesis. Taken together, it seems that at least in melanoma cells, the two extra-seed nucleotides in the mature miR-20a sequence render miR-20a into an inherent negative regulator of the miR-17/92 cluster.

## 5. Material and methods

### 5.1. Cells

The human cutaneous melanoma cell lines C8161 cells (HAG) and the PAG C81–61 were kindly provided by Dr Mary Hendrix (Children's Memorial Research Center, Chicago, IL, USA). PAG cells were grown in Ham's F10 medium supplement with 15% FBS, Pen/Strep and 1x MITO+ (BD Biosciences). HAG cells were grown in RPMI medium (Gibco/Invitrogen) supplemented with 10% FCS, Pen/Strep, l-Glutamine, Hepes and sodium pyruvate. HEK 293T cells (ATCC) were maintained in DMEM (Gibco/Invitrogen) containing 10% FBS (DMEM/FBS).

### 5.2. Luciferase reporter assay

C81–61 (PAG) cells were co-transfected with 1 μg of psiCheck2-ADAR1 3′ UTR (UTR), psiCheck2-ADAR1 mutated 3′ UTR (UTR-MUT) or psiCheck2-empty vector (No-UTR) and 0.1 μg of the pQCXIP-miR-17 (miR-17-5p) or pQCXIP-empty vector (mock) as control. HEK 293T cells were co-transfected with 1 μg of psiCheck2-ADAR1 3′ UTR plasmids (UTR), different psiCheck2-ADAR1 mutated 3′ UTR (UTR-mutA, UTR-mutB and UTR-mutAB) or psiCheck2–empty vector (No-UTR) and 0.1 μg of the pQCXIP-miR-20a (miR-20a) or pQCXIP-empty vector (mock) as control. Cells were harvested 48 h after transfection and assayed with Dual Luciferase Reporter Assay System (Promega) according to the manufacturer's instructions.

### 5.3. RNA isolation

Total RNA was isolated with Tri Reagent (Sigma-Aldrich) according to the manufacturer's instructions.

### 5.4. Quantitative PCR

First strand synthesis was executed using Universal cDNA synthesis kit (Exiqon) according to the manufacturer's instructions. Owing to its superior sensitivity, the SYBR Green master mix and specific-miRNA (miR-17 and miR-20a) LNA primers (Exiqon) were used according to the manufacturer's instructions for microarray validation and detection of specific miRNAs. Detection was carried out using the LC480 qPCR machine (Roche) according to the manufacturer's guidelines, followed by melting curve analysis at the end of the run.

### 5.5. Microarray expression analysis

Total RNA was extracted and used as template to generate cDNA and subsequent biotinylated target cRNA that was processed by an AffymetrixGeneChip Instrument System (Affymetrix) according to the manufacturer's recommendations (http://affymetrix.com/support/technical/manual.affx). The differentially expressed genes data were analysed by Ingenuity Pathway Analysis (http://www.ingenuity.com). The microarray data were deposited at NCBI GEO archives.

### 5.6. Cloning of pre-miRNAs

Genomic DNA was extracted from cells with the Wizard Genomic DNA Purification Kit (Promega). miRNAs were amplified with PCR from genomic DNA using specific primers (electronic supplementary material, table S3). Each amplified miRNA included the flanking genomic sequences of 110 bp from both sides. The amplicon was cloned into the pQCXIP vector (CloneTech) using the NotI and EcoRI restriction enzymes (New England BioLabs). Empty pQCXIP served as negative control. All cloned constructs were fully sequenced.

### 5.7. Cell transduction

A total of 2 × 10^5^ 293T cells were seeded in a 6-well plate and cultured overnight in DMEM (Gibco/Invitrogen) containing 10% FBS (DMEM/FBS). On day 1, cells were transfected with a mixture of 1 μg GAG-POL, 1 μg Envelope, 2 μg of each of the pQCXIP constructs and 6 μl of Turbofect reagent (Fermentas). After 6 h of incubation at 37°C, the cells were washed and re-cultured in fresh DMEM/FBS. On day 2, 5 × 10^4^ melanoma cells were placed in each well of 6-well plates and cultured overnight in DMEM/FBS. On day 3, the melanoma cells were infected with 6 ml of 0.45 μm filtered virion-containing medium of the 293T cells. After incubation at 37°C for 6 h, the infected melanoma cells were washed and re-cultured with fresh DMEM/FBS. The aforementioned infection procedure was repeated the next day on the same melanoma culture. Forty-eight hours after the second infection, selection was performed by addition of 1.2 μg ml^−1^ puromycine into culture medium.

### 5.8. Cell transfections

Cells were transfected with miR-17, miR-20a or scrambled sequence mimetics (Sigma Aldrich) supplemented with Turbofect reagent (Fermentas) according to the manufacturer's instructions.

### 5.9. Net cell proliferation

Melanoma cells (3 × 10^3^) were seeded in triplicate wells in 96F-well microplates. Net proliferation was determined by XTT colorimetric assay (Biological Industries), according to the manufacturer's instructions. Following background subtraction, the O.D. values were transformed into viable cell counts according to the specific regression equation that was determined for each cell line tested.

## Supplementary Material

Table S1

## Supplementary Material

Table S2

## Supplementary Material

Table S3
