# Supplementary material for: Differential regulation of aggressive features in melanoma cells by members of the miR-17-92 complex
Source: Open Biol. 2014 Jun 11;4(6):140030. doi: 10.1098/rsob.140030 (PMC4077061; doi:10.1098/rsob.140030)
Supplement: Table S3 [file rsob-14-0030-File008.doc]

**Table S3.** *List of primers*

|  | Forward | Reverse |
| --- | --- | --- |
| miRNA-17 | gtacgcggccgcgctgaatttgtatggtttatagttgtta | gtgaattcgcaccttagaacaaaaagcact |
| miRNA-20a | GTACGCGGCCGCGCATAGTTGCACTACAAGAAGAATG | GTGAATTCCCATAGAACAGTGTTCAGTAACAGG |
